# Occult renal cell carcinoma manifesting with epistaxis in a woman: a case report

**DOI:** 10.1186/1752-1947-5-79

**Published:** 2011-02-24

**Authors:** Georgios Fyrmpas, Ade Adeniyi, Simon Baer

**Affiliations:** 1 ENT Department, Conquest Hospital, The Ridge, St Leonards-on-Sea, East Sussex, TN37 7RD, UK; 2 Urology Department, Conquest Hospital, The Ridge, St Leonards-on-Sea, East Sussex, TN37 7RD, UK

## Abstract

**Introduction:**

Metastatic disease in the sinonasal region occurs rarely and the primary site may be elusive. This case highlights the possibility of an occult renal tumor manifesting with nasal symptoms and the risk of severe bleeding following nasal biopsy.

**Case presentation:**

We report the case of a 79-year-old Caucasian woman who presented with a six-week history of intermittent left-sided nosebleeds. She was fit, without previous surgery or anticoagulation. Nasal endoscopy and computed tomography showed a hemorrhagic mass occupying her left ethmoid cells and middle meatus. After a highly hemorrhagic biopsy, the lesion was histologically confirmed as clear cell carcinoma. Screening revealed a right kidney mass with widespread metastases. Palliative radiotherapy to the sinonasal metastasis and systemic treatment rendered her free of symptoms nine months after initial presentation.

**Conclusions:**

General practitioners and ear, nose and throat (ENT) doctors are very often confronted with epistaxis. A small minority of patients with epistaxis show a primary or metastatic nasal mass. Detection of the origin of secondary sinonasal masses requires a high index of suspicion and examination of infraclavicular sites by a multidisciplinary team. Renal cell carcinoma metastases are prone to severe bleeding during any surgical intervention, therefore, preoperative embolization is recommended. Resection or radiotherapy to the sinonasal metastasis of renal origin is justified in order to prevent recurrent nosebleeds.

## Introduction

Epistaxis is a common complaint that usually responds to conservative measures. Failure to control epistaxis after coagulopathies have been excluded should raise the suspicion of a nasal tumor. Nasal malignant tumors are usually primary and account for 0.3% of all neoplasms and 3% of all head and neck neoplasms [1]. Occasionally metastatic sinonasal tumors from infraclavicular sites, mainly the kidneys and, to a lesser degree, the lungs and breast, may manifest with nasal symptoms [2]. Up to the present 105 cases of maxillary metastases and 21 cases of ethmoid metastases from renal carcinomas have been reported [3].

The aim of this report is to describe a rare case of occult renal cell carcinoma (RCC) presenting with massive epistaxis due to a nasal cavity-ethmoid metastasis. The diagnostic difficulties and the current treatment options for metastatic renal cell carcinoma to the sinonasal region will be briefly discussed.

## Case presentation

**Figure 1:**
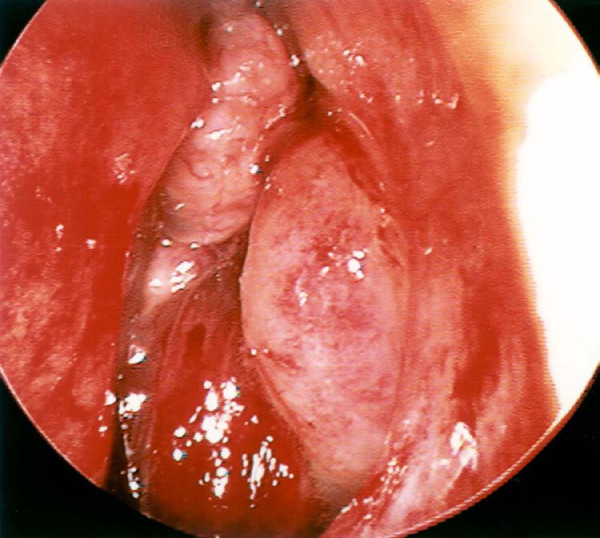
**Endoscopic view of hemorrhagic lesion protruding through the left middle meatus**.

**Figure 2:**
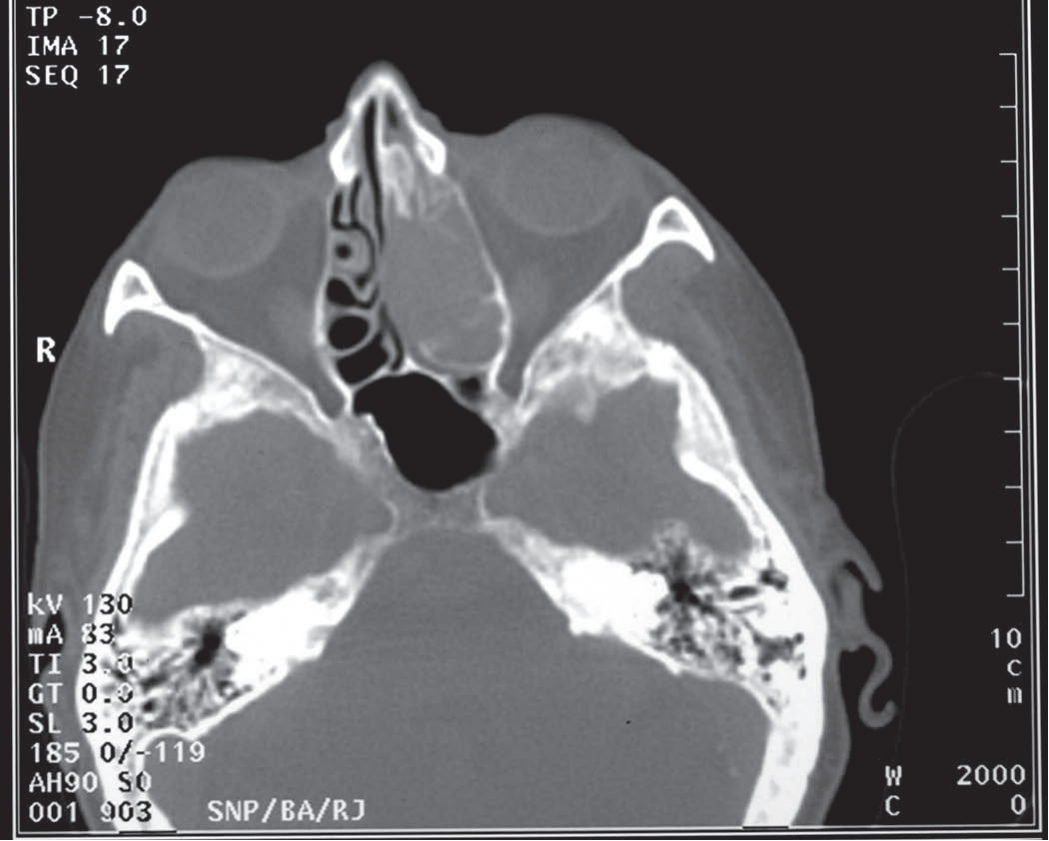
**Axial CT scan of the nose and paranasal sinuses showing that the lesion occupies the left nasal cavity and ethmoid sinuses**.

**Figure 3:**
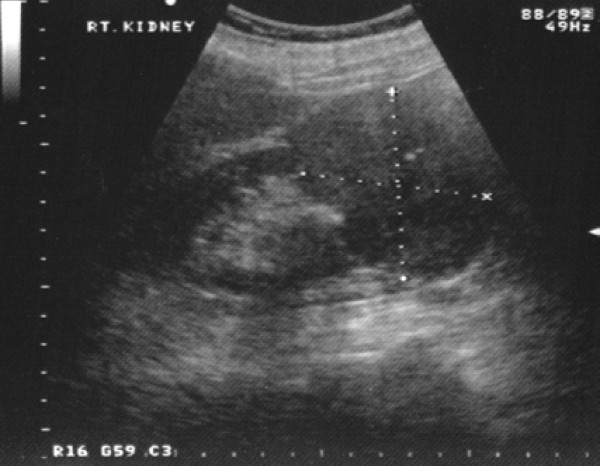
**Ultrasound examination reveals a large mass on the upper pole of the patients' right kidney**.

A 79-year-old Caucasian woman presented to our ENT department with a six-week history of recurrent progressive left-sided epistaxis. Her medical history was negative for hypertension, diabetes mellitus, surgery, bleeding tendencies and anticoagulation treatment. Laboratory tests showed marginally low haemoglobin levels (10 mg/dl) and normal calcium and lactate dehydrogenase (LDH) levels. On nasal endoscopy, a highly vascular mass arising from the left middle meatus was noted (Figure 1). Computed tomography (CT) of the nose and paranasal sinuses revealed an expanding mass in the left nasal cavity invading the ethmoids and extending to the floor of the left frontal sinus (Figure 2). A biopsy of the nasal mass under general anaesthesia resulted in profuse intra-operative bleeding, which necessitated anterior and posterior nasal packing. Histological examination of the specimen confirmed clear cell carcinoma of primary sinonasal or renal origin. A solid mass on the upper pole of the right kidney, measuring 65×63×99 mm, was noted on ultrasound examination (Figure 3). Surprisingly, urine examination was negative for haematuria. CT screening revealed widespread secondaries. Treatment with palliative radiotherapy and immunotherapy was instituted due to our patient's refusal of any interventional treatment. She remains asymptomatic nine months after initial diagnosis.

## Discussion

RCC grows slowly and becomes manifest after a considerable tumor size is reached. Therefore, many small and asymptomatic RCCs are incidentally detected on ultrasound examination for other conditions. Thirty percent of patients present with a distant metastasis [4] and only 10% exhibit the classical presentation of the tumor with flank pain, palpable mass and gross haematuria [5]. Intermittent haematuria, however, may be present in 90% of patients [5]. The most common sites of distant metastases of RCC are the abdomen, lungs, brain, liver, adrenal glands and bones [6]. Supraclavicular metastases usually occur in the thyroid gland, brain and very rarely the nose and paranasal sinuses. RCC tumor cells spread to the sinonasal region via two potential haematogenous routes: a) the route that follows the inferior vena cava, lungs, heart and the maxillary artery, in which case concurrent lung or brain metastasis may be present, and b) the route through the communication of the avalvular vertebral venous plexus and the intracranial venous plexous; in such case the sinonasal region may be the only site of metastasis [7].

RCC comprises a histologically diverse group of solid tumors; the most common histological variant being the clear cell RCC (85%) [8]. This variant is associated with loss of function of the von Hippel Lindau gene which leads to upregulation of the hypoxia inducible factor (HIF) and, finally, increased function of the vascular endothelial growth factor (VEGF) [9]. The net effect of this chain of events is increased angiogenesis and vascularity of clear cell RCC and related metastases. Therefore, sinonasal metastases of RCC origin are characterized by a propensity for severe bleeding [10].

A differential diagnosis of nasal bleeding lesions should include angiofibromas, hemangiopericytomas, hemangiomas and other less vascular malignant lesions such as adenocarcinomas, melanomas and metastatic tumors from the breast and lungs. A paranasal sinus CT scan may provide some hints about the benign or malignant nature of the lesion, such as bone erosion and remodeling (signs of malignant and metastatic lesions), hypervascularity, expansion of the sphenopalatine foramen and pterygopalatine fossa (angiofibromas). Magnetic resonance imaging (MRI) shows the true extent of the lesion, infiltration of the skull base and leptomeningeal metastases. Biopsy of a suspicious nasal lesion is imperative to guide further workup, but severe hemorrhage may occur [11]. Some authors advocate selective embolization prior to tumor biopsy particularly if there is a known history of nephrectomy [10,12]. Biopsy of RCC nasal metastasis may prove non-diagnostic due to diffuse necrosis of the lesion so several attempts are sometimes necessary [11]. If the histological specimen shows clear cells, the abdomen should be investigated with ultrasonography and CT. Other sites prone to RCC metastasis, such as the lungs, brain and bone, should be screened with CT and bone scintigraphy, respectively.

Patients with metastatic RCC have a poor prognosis with a median survival of seven to 11 months [4]. However, the biological behavior of RCC is variable and prognosis depends on clinical, radiological, serological and histological factors. Tumor stage and grade, the presence of vascular invasion and capsular infiltration, microvessel density and tumor necrosis are important clinicohistological prognostic factors. Low performance status (70 or less in Karnofski's scale), thrombocytosis, and neutrophilia, one and a half times higher than normal levels of serum LDH, low hemoglobin, corrected serum calcium levels higher than 10 mg/dL are poor prognostic indicators[13].

Metastatic RCC is resistant to radiotherapy and chemotherapy although a variable response has been reported [4]. According to the The National Comprehensive Cancer Network practice guidelines for kidney cancer [14], patients with a resectable primary tumor and a single metastasis or post-nephrectomy patients who develop a metachronous metastasis may benefit from nephrectomy and metastasectomy or metastasectomy respectively. If the primary tumor is potentially resectable but multiple metastases coexist, cytoreductive nephrectomy and systematic therapy is likely to be of benefit. Interferon α, interleukin 2, temsirolimus, surutinib and bevacizumab are currently evaluated in therapeutic protocols. If the primary tumor is unresectable and the nasal metastasis causes epistaxis and visual disturbances, the patient may receive systemic therapy or resection or radiotherapy of the metastasis.

## Conclusion

Sinonasal lesions presenting with epistaxis are rare. RCC metastases to the nasal cavity and paranasal sinuses should be included in the differential diagnosis of nasal bleeding lesions. Biopsy and resection of such lesions may result in profuse bleeding and, therefore, pre-operative embolization is recommended. RCC sinonasal metastasis signifies advanced disease with compromised survival. Resection or radiotherapy of the nasal metastasis with palliative intent will improve quality of life and the choice of treatment modality depends upon the patient's physical status and preference.

## Consent

Written informed consent was obtained from the patient for publication of this case report and accompanying images. A copy of the written consent is available for review by the Editor-in-Chief of this journal

## Competing interests

The authors declare that they have no competing interests.

## Authors' contributions

GF participated in the clinical care of the patient in the ENT Department, performed the literature review and wrote the report. AA participated in the clinical care of the patient in the Urology Department, examined the urology literature and contributed to the discussion section of this report. SB was the leading consultant in the care of this patient; additionally, he supervised and corrected this report. All authors read and approved the final manuscript.
